# The beneficial effect of propolis during an eight-weeks of resistance training on TNF-α and IL-6 genes expression in the kidney tissue of female Wistar rats undergoing testosterone enanthate consumption

**DOI:** 10.1016/j.crphys.2025.100167

**Published:** 2025-10-06

**Authors:** Khadijeh Molaei, Sanaz Mirzayan Shanjani, Ali Gorzi, Yaser Kazemzadeh, Abdolali Banaeifar

**Affiliations:** a Department of Exercise Physiology, Islamshahr Branch, Islamic Azad University, Islamshahr, Iran; b Department of Sport Sciences, University of Zanjan, Zanjan, Iran; c Division of Clinical Physiology, Department of Laboratory Medicine, Karolinska Institutet, Stockholm, Sweden; d Department of Exercise Physiology, South Tehran Branch, Islamic Azad University, Tehran, Iran

**Keywords:** Propolis, Testosterone enanthate, Inflammation, Cytokines, Kidney tissue, Resistance training, TNF-α, IL-6

## Abstract

This study investigated the potential benefits of propolis in mitigating the adverse effects of testosterone enanthate on the gene expression levels of pro-inflammatory cytokines, tumor necrosis factor-alpha (TNF-α), and interleukin-6 (IL-6) in the kidney tissue of female Wistar rats undergoing resistance training. Testosterone enanthate, a commonly used anabolic steroid among female athletes, is known to impair kidney function through inflammatory and oxidative pathways. Twenty-four female Wistar rats (8 weeks old, weighing 208.22 ± 14.17 g) were obtained from the Pasteur Institute. After a one-week acclimatization period, the rats were randomly divided into three groups: 1) training + placebo (n = 8), 2) training + testosterone enanthate (n = 8), and 3) training + testosterone enanthate + propolis (n = 8). The 8-week resistance training program involved a 1-m vertical ladder with 26 steps and additional weights. Training occurred five days a week with two rest days. Rats in the testosterone enanthate group received injections of 20 mg/kg body weight. The other group received the same testosterone supplementation plus propolis via gavage at 400 mg/kg body weight, three times a week. TNF-α gene expression in the training + testosterone group was significantly higher than in the training + placebo (*P* < 0.0001) and training + testosterone + propolis (*P* = 0.0025) groups. TNF-α gene expression in the training + testosterone + propolis group was also significantly higher than in the training + placebo group (*P* = 0.0001). Similarly, IL-6 gene expression in the training + testosterone group was significantly higher than in both the training + placebo (*P* < 0.0001) and training + testosterone + propolis (*P* = 0.0142) groups. IL-6 gene expression in the training + testosterone + propolis group was significantly higher than in the training + placebo group (*P* = 0.0016). These findings suggest that propolis supplementation can partially attenuate the inflammatory effects of testosterone enanthate on kidney tissue, likely through modulation of cytokines gene expression, highlighting its potential therapeutic role in preserving kidney health during anabolic steroid exposure.

## Highlights

• Investigates the anti-inflammatory effects of propolis on kidney tissue during resistance training.
• Examines TNF-α and IL-6 gene expression in female Wistar rats.
• Demonstrates that testosterone enanthate increases inflammatory cytokine levels.
• Shows that propolis supplementation reduces inflammation, though not to baseline.
• Provides insights into natural interventions for anabolic steroid-induced inflammation.

## 1 Introduction

Anabolic steroids are widely used to enhance physical performance by increasing strength, endurance, and muscle hypertrophy (Kicman and Gower, 2003). Among these, testosterone enanthate a synthetic ester of the natural androgen testosterone is particularly recognized for its effectiveness in promoting lean body mass and physical power (Masterson et al., 2020). Although often used in athletic settings, particularly among females engaged in resistance training, high doses of testosterone enanthate raise serious concerns regarding androgenic effects such as virilization, menstrual disturbances, and long-term endocrine imbalance (Sagoe et al., 2014). Beyond hormonal side effects, testosterone exerts systemic influence on vital organs like the kidneys, which regulate fluid and electrolyte balance, eliminate waste, and control blood pressure (Filho et al., 2020; Kovesdy, 2022). Importantly, the kidneys are highly responsive to systemic inflammation. Testosterone can increase the gene expression of pro-inflammatory cytokines such as tumor necrosis factor-alpha (TNF-α) and interleukin-6 (IL-6), which are known mediators of glomerular and tubular injury (Petreski et al., 2021; Tuttle et al., 2022). These cytokines activate NF-κB signaling and other pro-apoptotic cascades, promoting fibrosis, oxidative damage, and apoptosis of kidney epithelial cells.

Notably, IL-6 has been implicated in accelerating kidney hypertrophy and mesangial expansion, while TNF-α can induce podocyte injury and contribute to proteinuria. Dysregulation of these pathways can lead to both acute kidney injury and chronic kidney disease progression. Resistance training, while beneficial for muscle development and metabolic health, represents a form of physiological stress that elevates inflammatory signaling and oxidative load, especially when combined with anabolic steroids. This interaction may amplify kidney inflammation and injury. Moreover, female animals particularly female Wistar rats have shown higher sensitivity to testosterone-induced organ stress, making them a suitable model for studying the combined effects of exogenous androgens and exercise (Filho et al., 2020; Petreski et al., 2021). To mitigate such effects, natural supplements with antioxidant and anti-inflammatory properties are gaining attention. Propolis, a resinous substance collected by honeybees from plant exudates, is rich in polyphenols, flavonoids, and phenolic acids, and has demonstrated antimicrobial, antioxidant, and immunomodulatory properties (Almuhayawi, 2020; Forma and Bryś, 2021). These constituents downregulate NF-κB and phospholipase A2 activity, suppressing TNF-α and IL-6 expression.

Propolis also scavenges reactive oxygen species (ROS), regulates nitric oxide (NO) production, and alters macrophage polarization toward the M2 anti-inflammatory phenotype. Studies have demonstrated the potential benefits of propolis for kidney health. Silveira et al. (2019) demonstrated that 500 mg/day of propolis improved kidney function over 12 months in chronic kidney disease patients. Chermut et al. (2023) reported reduced kidney inflammatory markers after eight weeks of 100 mg/day oral supplementation. A systematic review by Zulhendri et al. (2021) further confirmed its capacity to attenuate oxidative and inflammatory damage in kidney and other tissues. Despite these promising findings, this study is the first to examine the triad interaction between testosterone, resistance training, and propolis on kidney inflammation in a controlled animal model. Therefore, this study investigates whether propolis supplementation can attenuate testosterone enanthate-induced elevation in TNF-α and IL-6 gene expression in the kidney tissue of female Wistar rats undergoing an eight-week progressive resistance training protocol. Findings from this study may inform therapeutic strategies for female athletes using performance-enhancing substances.

## 2 Methodology

### 2.1 Materials

***Animals***: Twenty-four female Wistar rats (8 weeks old, mean weight 208.22 ± 14.17 g) were obtained from the Pasteur Institute, Iran. Following a one-week acclimatization period, the rats were randomly divided into three groups: 1) Training + Placebo (n = 8); 2) Training + Testosterone Enanthate (n = 8); and 3) Training + Testosterone Enanthate + Propolis (n = 8). The rats were housed in transparent polycarbonate cages (15 x 15 × 30 cm; Razi Rad Company, Iran) in a controlled laboratory environment. Temperature was maintained at 22 ± 2 °C, with a humidity level of 45 ± 5 %, 12/12 h light/dark cycle, and adequate ventilation. The rats received pellet food, supplied by Karaj Behparvar Animal Feed Company in Iran, and had ad libitum access to water in a dedicated 500-ml container designed for laboratory animals. Two kidney tissue samples in the placebo group were excluded due to proteolysis during tissue handling. This was transparently reported and is consistent with accepted norms in similar experimental studies.

***Training Protocol***: In this study (ethics code: IR.IAU.TMU.REC.1399.347) experimental rats underwent an eight-week resistance training program (Meldrum et al., 2003) using a 1-m ladder with 26 steps and weighted loads. The resistance training protocol is presented in Table 1 with more details. The training consisted of five workout days and two rest days per week (Two weigh-in sessions were conducted per week; Banaeifar et al., 2011).

**Table 1:** Resistance training protocol.

Variable/time (week)	W 1	W 2	W 3	W 4	W 5	W 6	W 7	W 8
Load (% body mass)	40 %	60 %	80 %	100 %	80 %	120 %	140 %	160 %
Repetitions within sets	6	4	5	6	4	4	5	6
Number of sets	1	2	2	2	2	4	4	4
Rest Intervals between reps (s)	15	15	30	30	15	30	30	30
Rest Intervals between sets (s)	0	60	90	90	60	100	120	120

Supplement and Steroid Administration:

• **Testosterone enanthate:** testosterone enanthate (Aburaihan Pharmaceutical Co., Iran) was administered injection intramuscularly at a dose of 20 mg/kg body weight, three times per week, for 8 weeks, following Joksimović et al. (2017).
• **Propolis:** Propolis extract (SinaBee Co., Iran) was administered via oral gavage at 400 mg/kg body weight, three times per week, for 8 weeks, based on Barakat et al. (2015).

***Gene Expression analysis***: To eliminate acute effects of the interventions, testosterone injection and propolis gavage, kidney tissue samples were collected 48 h after the last training session, injection, and gavage, following a 12-h fasting period. Animals were anesthetized via intraperitoneal injection of ketamine (30–50 mg/kg) and xylazine (3–5 mg/kg). Kidney tissues were collected, washed with physiological serum, immediately frozen in liquid nitrogen (−196 °C), and stored at −80 °C until analysis. Gene expression of inflammatory markers TNF-α and IL-6 was measured using real-time PCR.

Total RNA was extracted from frozen kidney tissue using RNX-Plus reagent (CinnaGen, Iran), and concentration and purity were assessed with a NanoDrop ND-1000 spectrophotometer. First-strand complementary DNA (cDNA) was synthesized using the RevertAid First Strand cDNA Synthesis Kit (Thermo Fisher Scientific, USA).

Quantitative real-time PCR was performed using SYBR Green PCR Master Mix (Ampliqon, Denmark) in a Rotor-Gene Q thermocycler (Qiagen, Germany). Each 20 μL reaction contained 10 μL SYBR Green Master Mix, 1 μL of each primer (10 μM), 2 μL of cDNA template, and 6 μL of nuclease-free water. GAPDH was used as the endogenous control gene. Primer sequences were designed using NCBI Primer-BLAST and synthesized by Metabion (Germany). The genes expression levels of TNF-α and IL-6 were calculated using the 2^–ΔCT^ method (Table 2).

**Table 2:** Primer sequences for target and reference genes.

Gene	Forward Primer (5′→3′)	Reverse Primer (5′→3′)
TNF-α	CCTCTCTCTAATCAGCCCTCTG	GAGGACCTGGGAGTAGATGAG
IL-6	AGTTGCCTTCTTGGGACTGA	TCCACGATTTCCCAGAGAAC
GAPDH	AGACAGCCGCATCTTCTTGT	CTTGCCGTGGGTAGAGTCAT

### 2.2 Analysis

The assumptions for parametric statistical analysis were first verified. Outliers were checked, and the normality of data distribution within groups was confirmed using the Shapiro–Wilk test. Homogeneity of variances was assessed using Levene's test. After ensuring these assumptions, one-way analysis of variance (ANOVA) was performed to evaluate between-group differences. When the main effect was significant, Bonferroni post hoc tests were applied for pairwise comparisons. All *p*-values were presented in italics and considered statistically significant at *p* < 0.05. Additionally, effect sizes were reported using eta squared (η^2^) and Cohen's *d* to indicate the magnitude of differences independent of sample size. All statistical analyses were conducted using SPSS software (version 27, IBM Corp., USA), and graphs were generated using GraphPad Prism (version 9.4.0.673). Data are expressed as mean ± standard deviation (SD).

### 2.3 Results

At the beginning of the study, all rats were healthy and active, and the number of samples was the same in different research groups. Throughout the 8-week of resistance training, no signs of morbidity or mortality were observed. All animals tolerated the resistance training protocol and injections well. Body weight increased in all groups, with mean weight gains of 58.26 ± 8.75 g in the Training + Placebo group, 62.19 ± 8.69 g in the Training + Testosterone group, and 52.40 ± 7.24 g in the Training + Testosterone + Propolis group.

The one-way ANOVA results revealed statistically significant differences in the mean gene expression levels of TNF-α (F(2,19) = 39.550, *P* < 0.0001, η^2^ = 0.81) and IL-6 (F(2,19) = 25.283, *P* < 0.0001, η^2^ = 0.73) in the kidney tissues of the three experimental groups. The effect size values (η^2^) for both markers exceeded the 0.14 threshold, indicating large and meaningful differences between the groups.

**Fig. 1:**
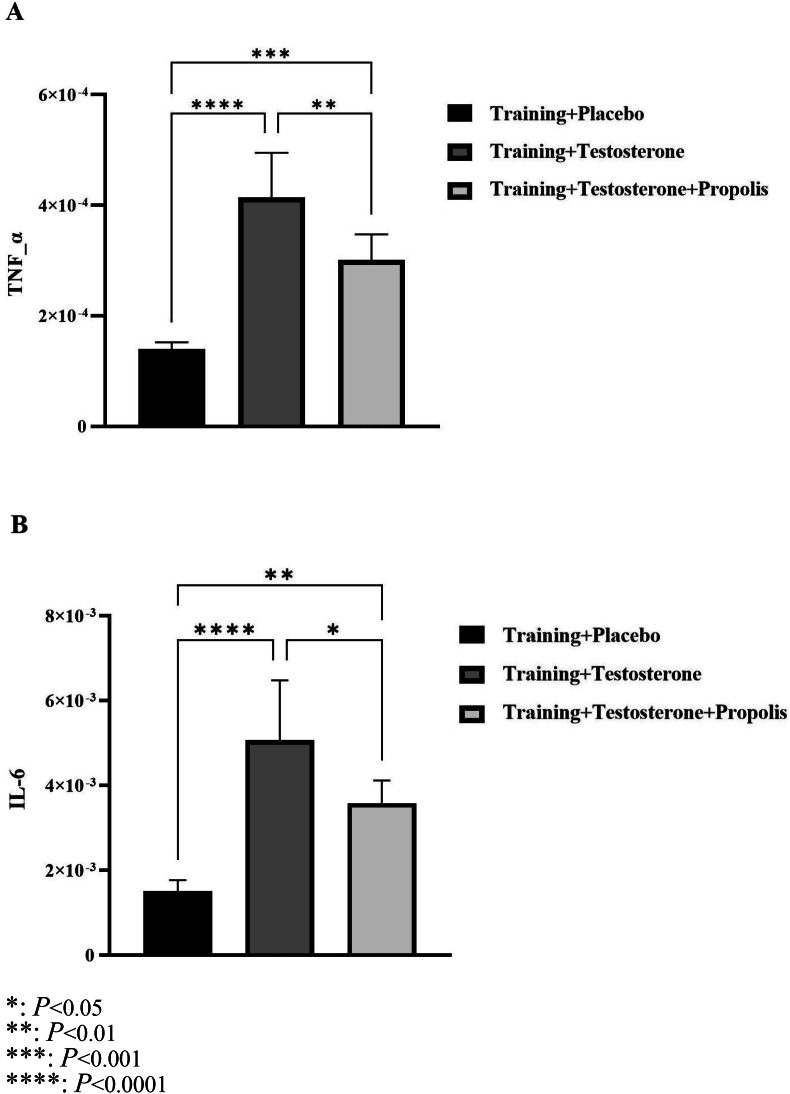
TNF-α (A) and IL-6 (B) gene expression levels in different studied groups. ∗: *P* < 0.05. ∗∗: *P* < 0.01. ∗∗∗: *P* < 0.001. ∗∗∗∗: *P* < 0.0001.

Bonferroni post hoc comparisons showed that the mean TNF-α level in the Training + Testosterone group was significantly higher than both the Training + Placebo group (Mean Diff. = 2.74 × 10^-4^, t(12) = 8.89, *P* < 0.0001, d_C_ = 4.40) and Training + Testosterone + Propolis (Mean Diff. = 1.13 × 10^-4^, t(14) = 3.96, *P* = 0.0025, d_C_ = 4.40) groups. Additionally, the Training + Testosterone + Propolis group had significantly higher TNF-α levels than the Training + Placebo group (Mean Diff. = 1.61 × 10^-4^, t(12) = 5.23, *P* = 0.0001, d_C_ = 1.71), (Fig. 1-A). Similarly, the IL-6 level in the Training + Testosterone group was significantly higher than both the Training + Placebo group (Mean Diff. = 3.56 × 10^-3^, t(12) = 7.11, *P* < 0.0001, d_C_ = 3.26) and the Training + Testosterone + Propolis group (Mean Diff. = 1.48 × 10^-3^, t(14) = 3.20, *P* = 0.0142, d_C_ = 4.66). The Training + Testosterone + Propolis group also exhibited significantly higher IL-6 levels than the Training + Placebo group (Mean Diff. = 2.08 × 10^-3^, t(12) = 4.15, *P* = 0.0016, d_C_ = 1.39), (Fig. 1-B).

All observed pairwise differences had d_C_ values greater than 0.8, confirming that the differences in inflammatory gene expression levels between groups were not only statistically significant but also of large magnitude.

According to Cohen's d interpretation, values equal to or greater than 0.8 represent large effect sizes. In this study, all calculated d_C_ values for pairwise comparisons of TNF-α and IL-6 levels exceeded this threshold, indicating strong and substantial differences between the groups. These findings confirm that the changes in cytokines levels are not only statistically significant but also biologically meaningful.

## 3 Discussion

The present study demonstrates that eight weeks of resistance training combined with testosterone enanthate administration resulted in a significant elevation in the gene expression levels of the pro-inflammatory cytokines TNF-α and IL-6 in kidney tissue. In contrast, when propolis was co-administered with testosterone, the gene expression levels of both markers were significantly reduced although they did not fully return to the levels observed in the placebo group. Thus, testosterone exacerbates kidney inflammation, whereas propolis provides partial protection against these effects.

The increased gene expression of TNF-α and IL-6 observed in the testosterone-treated group is consistent with prior studies. Metcalfe et al. (2007) reported that testosterone administration in rats with kidney obstruction elevated TNF-α levels, triggered apoptotic signaling, and promoted interstitial fibrosis, ultimately impairing kidney function. TNF-α plays a central role in mediating kidney damage, particularly under ischemic or obstructive conditions (Misseri et al., 2004, 2005), and facilitates immune cell recruitment and the amplification of other pro-inflammatory mediators (Guo et al., 1999, 2001). Furthermore, Park et al. (2005) demonstrated that testosterone interferes with protective signaling pathways, including nitric oxide synthase, Akt, and ERK, during kidney ischemia-reperfusion injury. Testosterone has also been shown to activate Toll-like receptors (TLRs), especially TLR4, on immune cells like macrophages, leading to the initiation of inflammatory cascades and the release of cytokines such as TNF-α and IL-6 (Sameer and Nissar, 2021; Hedger, 2011).

Mechanistically, testosterone enhances the activity of the nuclear factor kappa-light-chain-enhancer of activated B cells (NF-κB), a key transcription factor regulating the expression of inflammatory genes (Liu et al., 2017). It also contributes to macrophage polarization toward the pro-inflammatory M1 phenotype, thereby intensifying the immune response (Ren et al., 2017; Saqib et al., 2018). These pathways offer a plausible explanation for the inflammatory profile observed in the testosterone group. In contrast, the reduction in TNF-α and IL-6 gene expression in the training + testosterone + propolis group points to the therapeutic potential of propolis as an anti-inflammatory agent. Propolis is a natural resinous substance produced by bees, rich in flavonoids, phenolic acids, and terpenes, all of which possess immunomodulatory and antioxidant properties (Chermut et al., 2023; Silveira et al., 2019).

Several biological mechanisms underpin the anti-inflammatory effects of propolis. It inhibits NF-κB activation, thereby reducing the transcription of pro-inflammatory cytokines (Zulhendri et al., 2022). It also downregulates phospholipase A2 (PLA2), an enzyme central to the arachidonic acid cascade, which leads to reduced synthesis of inflammatory prostaglandins and leukotrienes (Sharma et al., 2007). Additionally, propolis acts as a potent antioxidant by scavenging reactive oxygen species (ROS), thereby mitigating oxidative stress that fuels chronic inflammation (Murakami and Kudo, 2002; Liu et al., 2023).

Propolis also helps regulate nitric oxide (NO) levels by modulating inducible nitric oxide synthase (iNOS) activity. While basal NO is essential for immune regulation, its excessive production can exacerbate inflammation; thus, propolis contributes to redox balance and immune homeostasis (Xu et al., 2022). At the cellular level, propolis promotes macrophage polarization from the M1 (pro-inflammatory) to the M2 (anti-inflammatory) phenotype, helping restore immune equilibrium (Alanazi et al., 2020). Moreover, its interference with intracellular signaling cascades adds to its broad anti-inflammatory potential.

In summary, propolis appears to mitigate testosterone-induced kidney inflammation through several interconnected pathways, including NF-κB and PLA2 inhibition, antioxidant action, nitric oxide modulation, and immune cell phenotype regulation. Although cytokine levels did not fully normalize, these findings support the potential of propolis as a complementary therapeutic approach to attenuate inflammation resulting from anabolic steroid use. Further investigations are needed to optimize dosage strategies and evaluate long-term impacts.

However, certain limitations should also be considered in this study. This study was conducted exclusively on female Wistar rats, with a relatively small sample size and fixed doses of testosterone enanthate and propolis, only two inflammatory markers were investigated, and the intervention period was limited to eight weeks. Therefore, the generalizability of the results to other populations or longer durations and different doses remains uncertain. Additionally, the lack of investigation into histopathological changes in kidney tissue can be noted.

## 4 Conclusion

This study highlights that testosterone significantly increases the pro-inflammatory cytokines TNF-α and IL-6, while co-administration of propolis exerts a partial but significant anti-inflammatory effect. These results have important implications for athletes engaged in resistance training and for bodybuilding populations using steroids. Overall, according to the findings of the present study, it appears that propolis consumption, due to its antioxidant and anti-inflammatory compounds, reduces the adverse effects of testosterone misuse on the kidney tissue of female rats. However, optimal strategies for the timing, dosage, and duration of antioxidant supplementation require further research to fully realize their benefits and elucidate the underlying mechanisms. It is also suggested that in future research, to ensure greater confidence in the results, histopathological changes in the tissue should be assessed as well. It appears that clarifying the interaction between exercise-induced adaptations, hormonal modulation, and phytotherapy agents like propolis can open new avenues for preventive and therapeutic strategies in sports medicine, nephrology, and metabolic health.

## Credit author statements

Khadijeh Molaei: Conceptualization, Methodology, Software, Validation, Formal analysis, Investigation, Data Curation, Writing original draft, Writing-Review & Editing. Sanaz Mirzayan Shanjani: Supervision. Ali Gorzi: Supervising, Writing-Review & Editing. Yaser Kazemzadeh: Supervision. Abdolali Banaeifar: Supervision.

## Declaration of competing interest

The authors declare the following financial interests/personal relationships which may be considered as potential competing interests: Sanaz Mirzayan reports was provided by Islamic Azad University Eslamshahr Branch. Sanaz Mirzayan reports a relationship with Islamic Azad University Eslamshahr Branch that includes: board membership. If there are other authors, they declare that they have no known competing financial interests or personal relationships that could have appeared to influence the work reported in this paper.

## Data Availability

Data will be made available on request.
